# Metronidazole-Induced Neurotoxicity: A Case Report

**DOI:** 10.7759/cureus.102617

**Published:** 2026-01-30

**Authors:** Inês L Caetano, Ana C Cunha, Guiomar Pinheiro, Sónia Chan, Luísa S Pinto

**Affiliations:** 1 Department of Infectious Diseases, Unidade Local de Saúde de Santo António, Porto, PRT; 2 Department of Nephrology, Unidade Local de Saúde de Santo António, Porto, PRT; 3 Department of Internal Medicine, Unidade Local de Saúde de Santo António, Porto, PRT

**Keywords:** adverse event, cerebellar dysfunction, hepatic abscess, metronidazole, neurotoxicity

## Abstract

Metronidazole, a commonly prescribed antibiotic to treat anaerobic and some protozoal infections, can lead to the development of serious, albeit rare, neurological toxicity, particularly in the setting of prolonged treatment and high cumulative dose.

We describe the case of a female patient in her 70s hospitalized for a hepatic abscess who received an extended course of metronidazole and subsequently developed confusion, prostration, dysarthria, ataxia, dysmetria, tremor of the head and upper limbs, diplopia, and nystagmus. Given the suspicion of an adverse drug reaction, metronidazole was discontinued, resulting in progressive neurological improvement. Brain MRI revealed a T2- and T2-fluid-attenuated inversion recovery (FLAIR) hyperintense lesion involving the splenium of the corpus callosum, compatible with metronidazole-induced neurotoxicity in this clinical setting.

This case highlights that, despite being uncommon, metronidazole-induced neurotoxicity should be considered in patients who develop new neurological symptoms during treatment with this drug, promoting timely recognition.

## Introduction

Metronidazole is a widely used antimicrobial agent with activity against anaerobic bacteria and several protozoa. Among the most common indications for its use are pyogenic abscesses, including hepatic abscess, typically as part of extended antibiotic regimens, often lasting several weeks [1,2]. Metronidazole is a nitroimidazole prodrug that is activated under low-oxygen conditions in susceptible microorganisms, generating reactive compounds that damage DNA and lead to cell death [3].

Exposure to metronidazole has been associated with rare cases of neurotoxicity through mechanisms that remain incompletely understood [1,2]. Although uncommon, this adverse event warrants prompt recognition, as delayed identification can lead to significant neurological morbidity. Neurotoxicity usually manifests weeks after antibiotic initiation and is more frequently associated with prolonged exposure and higher cumulative doses [2,4,5]. The most common clinical findings are peripheral neuropathy, cerebellar dysfunction with ataxic gait and dysarthria, visual impairment, vestibulotoxicity, cochleotoxicity, and seizures [2,6]. These manifestations typically resolve partially or completely after withdrawal of the drug [2,5]. Because symptoms are nonspecific and may develop insidiously, diagnosis can be challenging [2].

Magnetic resonance imaging (MRI) signal abnormalities are frequently reported in this condition, including symmetric hyperintensities in T2- and T2-fluid-attenuated inversion recovery (FLAIR)-weighted sequences, often with restricted diffusion on diffusion-weighted imaging (DWI), predominantly located in the cerebellar dentate nuclei bilaterally, although involvement of the corpus callosum and brainstem may also occur [2,7].

This report describes a case of metronidazole-induced neurotoxicity, with predominance of cerebellar symptoms, in a patient undergoing prolonged therapy for a hepatic abscess.

This article was previously presented as a poster at the 27th Portuguese National Congress of Internal Medicine on October 4, 2021.

## Case presentation

**Figure 1:**
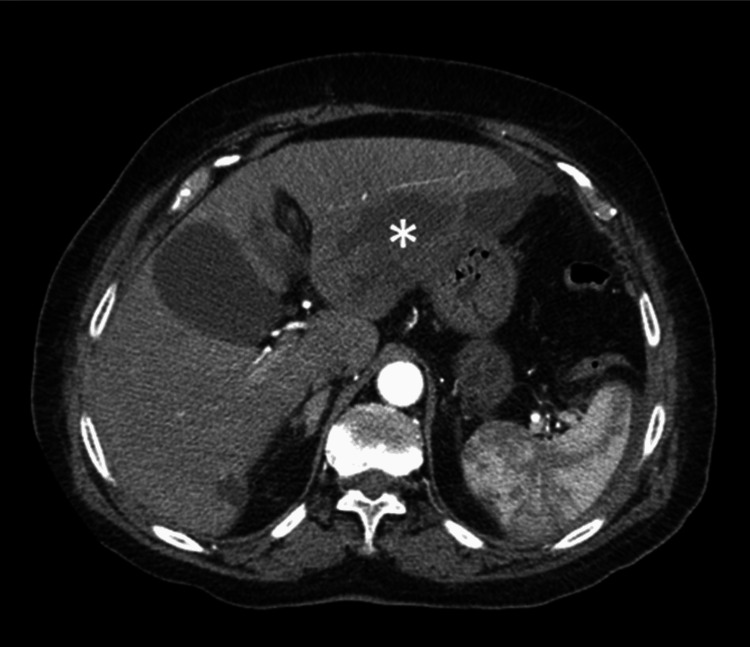
Abdominal computed tomography scan showing a large multiloculated abscess in the left hepatic lobe (asterisk)

­A female patient in her 70s, with a prior medical history of hypertension, dyslipidemia, obesity, moderate aortic stenosis, peripheral venous insufficiency, and nephrolithiasis, was admitted to the emergency department with a one-week history of abdominal pain in the upper quadrants, fever, and anorexia. At the time of admission, she had been receiving amoxicillin/clavulanate for six days for a suspected urinary tract infection. On presentation, the patient was afebrile and hemodynamically stable. Physical examination revealed generalized discomfort on abdominal percussion and pain on palpation of the upper quadrants, without signs of peritoneal irritation; the remainder of the examination was unremarkable. Blood counts and biochemical laboratory tests showed leukocytosis (25,300 cells/µL) with neutrophilia (22,720 cells/µL), an elevated C-reactive protein (170 mg/L), and acute kidney injury (creatinine 1.41 mg/dL and urea 73 mg/dL), while liver function tests were within normal limits. An abdominal computed tomography (CT) scan was performed, displaying a large multiloculated abscess in the left hepatic lobe (7 × 7 cm), with perihepatic ascites (Figure 1).

The patient was admitted to the hospital, and empiric antibiotic therapy with ceftriaxone and metronidazole (500 mg intravenously every six hours, corresponding to a daily dose of 2 g) was started. Due to persistent fever, worsening abdominal pain, and a rise in inflammatory markers, treatment was escalated after four days to piperacillin-tazobactam while maintaining metronidazole. Blood cultures obtained at admission prior to antimicrobial therapy and again before escalation were sterile. Percutaneous drainage of the hepatic abscess with catheter placement was performed on hospital day 10, with imaging reassessment three days later showing marked but incomplete reduction in abscess volume, which prompted placement of a second catheter to drain the associated perihepatic ascites. Abscess pus and ascitic fluid were sent for bacterial (aerobic and anaerobic) and mycobacterial studies, all of which showed no microbial growth. Following antibiotic escalation and drainage, the patient demonstrated progressive clinical and laboratory improvement regarding the infectious process, and both drains were subsequently removed 10 days after the initial placement. However, during hospitalization, she continued to experience nausea, vomiting, and anorexia, leading to malnutrition and significant weight loss (19% of her usual body weight). Given incomplete abscess drainage, a prolonged course of antimicrobial therapy with piperacillin-tazobactam and metronidazole was continued with planned radiological reassessment; metronidazole was maintained intravenously due to gastrointestinal intolerance.

On day 33 of antibiotic therapy, the patient developed episodes of confusion and prostration, with progression by day 37 to a predominantly cerebellar syndrome. At symptom onset, she was afebrile and hemodynamically stable, with normal renal and hepatic function and no metabolic disturbances. Neurological examination revealed spontaneous downbeat nystagmus in primary position and all gaze directions, scanning dysarthria, head and upper limb tremor, marked bilateral dysmetria (right-sided predominance), postural instability with retropulsion, and intermittent horizontal diplopia; motor strength and sensation were preserved. A head CT scan showed no evidence of acute intracranial pathology.

Given the subacute evolution of neurological symptoms in the context of prolonged metronidazole exposure, metronidazole-induced neurotoxicity was suspected. Wernicke’s encephalopathy was also considered due to malnutrition, and thiamine supplementation was initiated; however, vitamin B1 levels were within normal limits. Metronidazole was discontinued on day 37, after a cumulative exposure of approximately 74 g, followed by neurological improvement within 48 hours and gradual recovery thereafter with supportive care and rehabilitation.

**Figure 2:**
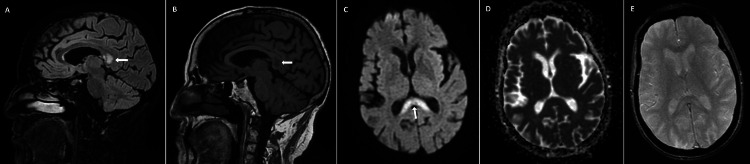
Brain magnetic resonance imaging performed 10 days after metronidazole discontinuation (A) Sagittal T2-fluid-attenuated inversion recovery (FLAIR) image showing a hyperintense lesion involving the splenium of the corpus callosum (white arrow); (B) sagittal T1-weighted image demonstrating hypointensity of the lesion (white arrow); (C) axial diffusion-weighted imaging (DWI) showing hyperintensity of the lesion (white arrow); (D) axial apparent diffusion coefficient (ADC) map showing no evidence of restricted diffusion; (E) axial T2*-weighted gradient-echo (GRE) image demonstrating no susceptibility changes

Brain MRI performed 10 days after metronidazole withdrawal demonstrated a hyperintense lesion involving the splenium of the corpus callosum on T2- and T2-FLAIR-weighted sequences (Figure 2A), with corresponding hypointensity on T1-weighted images (Figure 2B). DWI showed hyperintensity of the lesion (Figure 2C) without restricted diffusion on the apparent diffusion coefficient (ADC) map (Figure 2D). T2*-weighted gradient-echo (GRE) sequences revealed no susceptibility changes suggestive of hemorrhage (Figure 2E). Contrast-enhanced sequences were not performed.

At the time of hospital discharge, approximately one month after metronidazole discontinuation, the patient had achieved complete neurological recovery, which was subsequently confirmed at a follow-up outpatient visit two months later. No follow-up brain MRI was performed at the time of recovery.

## Discussion

Metronidazole is widely used in clinical practice, and although neurotoxicity is an uncommon adverse reaction, it has been increasingly reported. In this case, the neurological presentation was dominated by a subacute cerebellar syndrome, similar to the clinical phenotypes described in systematic reviews and individual case reports [2,5,8,9].

At the time of neurological deterioration, the patient had received a high cumulative dose of metronidazole (approximately 74 g) over a prolonged treatment period. Higher cumulative exposure has been associated with an increased risk of neurotoxicity, although similar manifestations have also been reported with shorter treatment courses and lower total doses [2,5,10]. Proposed risk factors include liver cirrhosis, chronic kidney disease, intravenous administration, and lower body weight; however, renal and hepatic functions were normal at symptom onset in our patient, indicating that metronidazole-induced neurotoxicity may occur in the absence of these predisposing conditions [11].

Brain MRI demonstrated a hyperintense lesion of the splenium of the corpus callosum on T2- and T2-FLAIR-weighted sequences, a pattern that has been described in association with metronidazole exposure but is not specific for this etiology and may be observed in a wide range of conditions within the spectrum of cytotoxic lesions of the corpus callosum, most frequently drug- or toxin-related, infectious, vascular, seizure-related, and metabolic [2,7,12]. Although restricted diffusion on DWI is commonly reported, its absence does not exclude the diagnosis of metronidazole-induced neurotoxicity, as diffusion abnormalities may be variable and reversible in toxic-metabolic encephalopathies [2,7].

Metronidazole-induced neurotoxicity can be challenging to recognize due to its subacute and nonspecific presentation. In the present case, differential diagnoses that may present with similar clinical or imaging findings, such as Wernicke’s encephalopathy, Marchiafava-Bignami disease, hepatic encephalopathy, posterior circulation stroke, and other drug-associated encephalopathies, were carefully considered [2,13]. However, the combination of the patient’s medical history, clinical evolution, laboratory tests, and neuroimaging findings was not consistent with these alternative diagnoses.

The mechanisms responsible for metronidazole-induced neurotoxicity remain uncertain, although several have been proposed [2]. Experimental data in animal models suggest that metronidazole crosses the blood-brain barrier and is associated with increased oxidative stress and neuroinflammatory responses mediated by reactive oxygen species, nitric oxide, and inflammatory cytokines, which may lead to neuronal injury [14]. An autopsy case report has described demyelination and axonal degeneration in a severe, irreversible presentation, indicating that structural damage may occur in some cases [15]. In addition, impairment of thiamine phosphorylation through the formation of a thiamine analog has been hypothesized as a potential mechanism, possibly contributing to a Wernicke-like encephalopathy [6].

A causal relationship between metronidazole and the neurological syndrome in our patient is suggested by the temporal association with prolonged exposure, the absence of alternative metabolic or structural causes, and the rapid clinical improvement following drug discontinuation, which is consistent with a probable adverse drug reaction, as well as neuroimaging findings compatible with metronidazole-induced neurotoxicity in this clinical context.

No specific treatment exists for metronidazole-induced neurotoxicity, and management relies on prompt discontinuation of the drug and supportive care. In most reported cases, the prognosis is favorable, with partial or complete improvement of clinical and imaging abnormalities after withdrawal, as observed in our patient [2,5,8]. Nevertheless, irreversible neurological deficits and even fatal outcomes have also been described, underscoring the importance of early identification of this condition [2].

## Conclusions

Metronidazole-induced neurotoxicity is a rare, underrecognized, and potentially severe adverse event. This report aims to alert clinicians to this entity and highlights the importance of maintaining clinical awareness for new neurological symptoms in patients treated with metronidazole, especially during prolonged courses and at high cumulative doses. Although conclusions from a single case must be interpreted with caution, early recognition and prompt drug cessation are essential to optimize outcomes.
